# What a mess: can we tidy up the concept of health?

**DOI:** 10.1080/09515089.2024.2369687

**Published:** 2024-06-19

**Authors:** Havi Carel

**Affiliations:** University of Bristol

**Keywords:** philosophy of medicine, concepts of disease, health, phenomenology of illness, Elizabeth Barnes

## Abstract

This is a review article of Elizabeth Barnes’ new book, *Health Problems*. In this article I try to offer a sense of where this exciting sub-discipline of philosophy of medicine has got to. I do that in three ways. First, I make a few comments on the general idea that there are theories of health competing in the field of philosophy of medicine; second, I offer specific comments on the phenomenological approach; and finally, I comment on Barnes’ claim that health is messy. I do not provide an overview of the book but a response to some of its themes.

## Introduction

*Health problems* is a good book. It is comprehensive, careful, and detailed in the way good philosophy is. It is clearly written and provides a useful overview of the state of theories about health and disease. If you want to know the lie of the land when it comes to concepts of health and disease, this is the book for you.

I’d like to say a little more not so much descriptively (other reviews already do that well) but to try and get a sense of where this exciting sub-discipline of philosophy of medicine has got to. I’ll do that in three ways. First, I’ll make a few comments on the general idea that there are theories of health competing in the field of philosophy of medicine; second, I’ll offer specific comments on the phenomenological approach; and finally, I’ll say something about Barnes’ claim that health is messy. I do not provide an overview of the book but a response to some of its themes. References that only give page numbers refer to *Health problems*.

### Theories of health?

The strategy deployed in the book is to survey existing philosophical theories of health, each with its strengths and weaknesses, in a succession of critiques that cumulatively leaves the stage empty and awaiting a new theory of health, as none of them are without problems. Barnes then introduces her own theory, which she calls ameliorative scepticism, in the book’s final two chapters.

The general idea is that health is a complex and messy concept with many overlaying components, strands and meanings that cannot be bundled into a neat philosophical account. No single theory, Barnes claims, can capture *everything* but also *only* what we mean when we talk about health. Hence – in what may be anathema to some philosophers – we ought to discard our attempt to achieve a single, unified theory of health in favour of a healthy scepticism. This scepticism is ameliorative in a Haslanger-style way: in part at least, the concept we strive towards ought to come out of asking not just what health is, but what it ought to be, given our social justice goals.

This strategy of presenting, criticising and then rejecting theories is repeated in the chapter on ameliorative scepticism, although this time it is directed at what she calls “traditional forms of metaphysical scepticism”: error theory, eliminativism, fictionalism, nonreductive realism, theories of vagueness and inconsistent objects, pluralism and pragmatism (p.192). Once explaining how each of these falls short or is unsuitable for the task at hand, namely that of providing a deflationary account of health, again the way is open to Barnes to lay out her positive view (which she calls ameliorative scepticism) in the book’s final two chapters. “I’m going to suggest” Barnes writes, “that we need a middle ground between traditional forms of metaphysical skepticism […] and various forms of realism” (p.192). This strategy is useful and informative and gives a fair hearing to the positions surveyed. Each position is presented – open sandwich style – by first outlining its strengths, then its weaknesses and ending with the judgement that the view is unsatisfactory and we need to search on.

I want to offer a few comments on this strategy, repeated twice in the book: once when surveying philosophical theories of health and once when surveying metaphysical scepticism. First, I think this strategy comes at the considerable cost of erasing much of the diversity and nuance of the views on offer. The theories that Barnes presents as ‘theories of health’ are in fact a mixed bunch of views; some are theories of disease, some of health, yet others try to offer analyses of the experience of illness. They have quite different target objects and goals. And they are not all aimed at offering a philosophical account of health. Barnes is aware of this, but I presume considers this a price worth paying to benefit from the advantages of a unified survey. In the beginning of Section 1.1, Barnes writes: “there is not a single body of literature here or a single shared set of terms or methodological assumptions”, and “there are many different debates, and different philosophical conversations, that can be seen as attempting to answer the question ‘what is health?’” (p.15). I think that retaining the diversity and different origins, goals, and methods of these views is paramount and should be held in mind whilst continuing to explore Barnes’ argument.

Second, Barnes’ strategy is also premised on the notion that there can only be one, or there must be a best, account of health. As I suggest below, by Barnes’ own lights (her ameliorative scepticism approach) there needn’t be and cannot be such an account; at best we choose the account that is best suited to our localised purposes (e.g. trying to understand the notion of health within sports medicine, trying to construct the fairest welfare system, and so on). I’m therefore not sure Barnes needs to reject all the accounts before setting out her own. A positive result of this strategy is that we are presented with a handy survey of philosophical approaches to health, provided in the introduction and in Chapter One. These include function-based accounts (sometimes called ‘naturalistic’ theories of disease), norm- or value-based approaches (usually referred to as ‘normativist’ approaches), hybrid theories (which aim to combine the two previous approaches), social constructionist views, and phenomenological accounts.

Regarding the first point on erasing the diversity of views. I think that the theories presented in Chapter One as ‘theories of health’ are not all about the same thing and describing them as such obscures this diversity. Some of these theories aim to develop an account of somatic disease; others might look for an account that includes mental disorder; yet others might have as their target the concept of health, trying to understand what health, not disease, is. These theories may, or may not, see health as a positive concept, over and above the absence of disease. This diversity raises several challenges to Barnes’ account in the early chapters of the book. First, it is not correct to say that all the accounts presented aim to provide an account of health. Christopher Boorse (1977), for example, offers what he calls a biostatistical theory of disease. Jerome Wakefield (1992) offers another account of disease, again, as ‘harmful dysfunction’. Phenomenological accounts and descriptions of illness vary. For example, Jean-Paul Sartre talks about illness as the experience of interruption and disturbance of an activity (e.g. a headache interfering with reading), and Maurice Merleau-Ponty (2012) describes illness as “a complete form of life”. Geroge Canguilhem (1991), in his proto-phenomenological account, contrasts what he calls ‘the normal’ and ‘the pathological’. Others distinguish between disease and illness (see below) in order to analyse illness, the experience of disease, but have little to say about disease or health. Lennart Nordenfelt (1987) offers an account of health, but Rachel Cooper (2002), a fellow normativist, offers an account of disease. This is complicated by the fact that further terms, e.g. illness and sickness, are also used to convey different aspects and dimensions of this complex thing Barnes calls ‘health’.

In terms of goals, there are probably no philosophers, or even philosophy straw-people, who think that we can give a list of necessary and sufficient conditions for something to count as disease, which will face no exception nor encounter challenging borderline cases. Each of the views presented in Chapter One will have ways in which the definition of health or disease it offers (if it offers one) is too narrow, so excludes important cases and too permissive, so includes important cases we’d ideally want to exclude. That, on its own, will not help us pick out the best theory because all of them are – and have been – extensively presented with counterexamples and problematic borderline cases.

For example, it has been noted that within Boorse’s (1977) biostatistical theory homosexuality would be a disease – a dysfunction harmful to the individual’s ultimate goal, that of reproductive success. But this is a view we would not endorse for moral and political reasons and which has therefore been presented in the literature as showing that Boorse’s biostatistical theory is not a robust account of disease, because it classifies as disease states we would not want to class as such. Under normativism, we would be obliged to admit that the same physiological condition – e.g. a skin disease that causes skin discolouration – might count as a disease in a dermatologist’s office in London but not in another place (or time, or culture), in which the distinctive patterns on the skin created by the disease are considered attractive. And so on.

Such examples are not, I think, on their own, reason enough to reject a view. It may be that the things we call disease are too diverse and different in important ways and hence as Barnes suggests, we ought to reject all of these attempts. However, another way to go about this is to reject the idea that we can only have one view of disease that would fit all contexts. It may be that we ought to use a naturalistic definition of disease for one set of conditions, e.g. somatic diseases, but require a normativist or social constructionist account to deal effectively with mental disorder. This approach – which we could call pluralism – is criticised by Barnes in Chapter Five but is not galaxies away from Barnes’ ameliorative scepticism presented in the final two chapters of the book.

It is also far from clear that all of these accounts of health and disease have the same goal. By Barnes’ own lights a concept will change depending on both the context in which it is used and also depending on the aims of the speaker using the concept (see her very interesting discussion in sections 4 and 5 of Chapter Six on Delia Graff Fara on shifting standards). Using that idea, we can look back on the critical chapters in which Barnes surveys accounts of health and disease, to see how her analysis of Fara’s account might work here. Barnes suggests, following Fara, that “we typically don’t have precise aims or purposes when we talk about health” and hence philosophical and conceptual discussions of health may inherit that imprecision and lack of specificity (p.239). We can modulate the specificity and hence the precision of our concepts when we evaluate health in a narrower (or broader) way, to suit our specific goals. If we accept this analysis, we ameliorate some of the worries about lack of precision: the lack of precision in debates on what health is, is not intrinsic to the concept of health, but arises from the differing context and goals we have when we talk about health (p.240).

As an important aside, it is often wrongly assumed that medical discourse and clinical assessments require precision. In fact, the opposite is often the case. Experienced clinicians often caution patients against getting too hung up “on the numbers” (e.g. percent of predicted lung function) or taking descriptors such as ‘moderate’ or ‘end stage’ too literally. In the context of Barnes’ proposal, it is important to be aware of this, as we tend to erroneously assume that precision is required for clinical talk but not to wellbeing talk. It may well be the opposite.

The important point comes, to my mind, in section 3 of Chapter Five, when Barnes claims that “we often benefit from having imprecise aims and goals and it would not always be better for us to make our aims and goals more precise” (ibid.). I agree with this claim and so want to suggest that perhaps normativists focus on the ways people in different cultures, with different norms, might understand health differently (and hence, possibly, normativists in general seek looser accounts), while naturalists try to provide a stringent definition that will apply only and always to instances of disease (and hence seek a tighter account). Barnes helpfully adds: “very often imprecise purposes are in fact fairly well adapted to suit imprecise reality” (p.241). The account is particularly apt when Barnes adds the next, and final element, that “sometimes we disagree with each other because we have different aims and goals” (ibid.). This, I think, sheds light on disagreements about the best definition of disease: it is, at least in part, a divergence in purposes, but these are often implicit and so hard to notice. Instead of presenting all the theories as uniform in their target object and goal, it would be useful to see the diversity of approaches as exemplifying Barnes’ view.

It would be right to point out that those providing arguments for and against particular accounts of disease are often arguing directly against their counterparts who hold alternative views. But that on its own does not prevent us from suggesting that the apparent disagreement rests, at least in part, on different goals: the different work each approach wants their account of disease to do.

Barnes seems to agree with this when in the introduction she discusses the fact that health has several different dimensions on which it is significant. These include biological, normative, political, and phenomenological dimensions. She claims that “there is a biological reality to health” (p.3) but also that health is morally significant: “it’s a way of being harmed” (ibid.) and it is “something we almost always value” (p.4). Health is “not just something we view as a personal good”; it is also something “we view as a public good” and is shaped by our public life (p.5). And finally, that health also has a “curiously distinctive phenomenological aspect” (ibid.). It is easy to see how emphasising one of these dimensions over others would yield a different account of health. And maybe that’s no bad thing. If we go down this route it may be that we do not need to adjudicate between the naturalists, normativists, hybrid theorists, etc. and can instead say that they might each have a different goal when developing their accounts.

Finally, it strikes me that the attempt to provide an account of health is considerably more ambitious, and of its nature more normative, than an attempt to define disease. Health can be understood minimally as absence of disease but intuitively most of us would think there is more to health than that. This is explored in the two chapters on wellbeing and capabilities in *Health Problems*, in which Barnes expands on this point.

### The three axes

Barnes proposes three axes along which theories of health can be positioned and classified: (1) negative/ positive; (2) evaluative/ nonevaluative; (3) subjective/ objective. I am not convinced the first and third help us understand the “jungle of theories” Barnes is trying to organise, present and assess in her first chapter, although the second axis certainly does.

**The negative/ positive axis** requires us to address a preliminary question, namely, which is the primary concept: is health simply the absence of disease (and then we need to define disease first), or is disease impaired health (and then we need to define health first)? This can get confusing.

I think it would do more justice to the theories to say that some take health as their primary target and others take disease, rather than try to unify them as all being theories of health. In addition, it is not clear that disease theories are negative theories of health, either (so define disease as ill health or the absence of health). Many views of health offer something beyond the absence of disease: flourishing, capabilities, wellbeing, etc. and these terms usually do not play a major role in discussions of disease definitions, nor are they needed in order to offer accounts of disease. Moreover, some theorists insist that the presence of disease is not mutually exclusive of flourishing or wellbeing. For example, the notions of ‘health within illness’ (Moch 1989) and of ‘ill, but well’ (Carel 2016, chapter 6; 2018, chapter 3) aim to capture precisely this point. I’m not sure such theories need to define health, or that their account should posit health as the primary concept, with disease derived from it in some way. Barnes’ assumption that all these theories are ultimately theories of health is neither justified nor necessary.

To complicate matters, there are also further distinctions offered in the literature, namely sickness (Twaddle 1992; Hofmann 2002) and illness (Carel 2016, 2018). Sickness was proposed in order to denote the social role of disease, or disease as entitling its bearer to certain social exemptions and social treatment. Illness is used for distinguishing between the physiological process (disease) and the experience of that process (the experience of disease, called illness). Barnes usefully proposes the term ‘pathology’ which I like, to capture disease, injury and impairment (in the same way Cooper (2002) defines disease as encompassing all of those). But the term ‘pathology’ fails to account for the important distinction between biological processes occurring in the body, and how such processes are felt and experienced. Hence the term ‘illness’ is crucial to us and other animals *qua* sentient beings (in human and veterinary medicine) when we prescribe medication intended to control pain, nausea, and dizziness, for example – symptoms that are experientially terrible although they may not be physiologically significant. More on this in the next section, where I discuss phenomenology of illness.

I’ll offer a final, more speculative comment before moving on to the second axis. When we accept the term ‘pathology’ we assume pathological states are cases of normality gone wrong, and in this sense they are derivative. This may well be the case in almost all cases; but let me offer one thought. There are conditions, such as autism spectrum disorder and ADHD, which until recently were thought of as dysfunctions, aberrations of normality, that are bad to have and as conditions that ought to be eradicated if possible. More recently neurodiversity proponents have claimed that minority cognitive styles are not defective forms of neurotypical brains, but simply different forms, and moreover, that this diversity is beneficial to us in important ways (Chapman 2021, 2023). I am *not* suggesting that central cases of pathology, like cardiac disease or lung cancer, are analogous, or good to have. Rather, I suggest that given that disease is intrinsic to human life as it currently stands, and given that medicine now allows people to co-exist for extended periods with a chronic condition (sometimes for many decades), we need a more nuanced account of illness, the experience of living with disease. Hence we may want to think of *some* disease states not as derivative to health (e.g. impaired health, absence of health) but as different ways of being. Not because they are recommended in any way, but because they are significant ways in which humans exist stably and in large numbers and as such merit investigating and developing a phenomenological language for. I am not saying these are good or better ways of being, but that they ought to be acknowledged and studied as complete human forms of life. While we can evaluate them as normatively negative (bad for us, we are unlucky to have them, as Rachel Cooper would say) we could still benefit conceptually, clinically, and morally from seeing them as ‘forms of life’ rather than merely and only as privative modes of health (Carel 2021b).

**The second axis, evaluative/ nonevaluative**, is the one that has garnered the most attention in the philosophy of medicine literature. Much space has been devoted to the question whether naturalist accounts of disease really are value-free, and whether value-ladenness is a negative and distorting feature of theories of disease. I won’t rehearse these here but say that in my view this is the important axis and bone of contention between naturalist and normativist views. I think Cooper (2002) succeeded in showing that no truly value-free account of disease is possible and gave a persuasive explanation of why not. As Cooper concludes, “no biological account of disease can be provided because this class of conditions is by its nature anthropocentric and corresponds to no natural class of conditions in the world” (2002, 271).

**The third axis, subjective/ objective**, seems to me the most problematic. It is not clear that you could have a viable theory at either end of the spectrum. No account of health (or of pathology) could be entirely subjective (I can’t be healthy, or ill, just in virtue of believing that I am). No account of human health (or of pathology) could be entirely objective (I can’t be healthy or ill without either state having an experiential dimension to it). It might be that instead of an axis, what is needed is a way to distinguish between the objective components of a health state (‘disease’ which refers to biological dysfunction) and the subjective or experiential components (‘illness’ which refers to the experience of the dysfunction). More on this distinction in the next section.

### On Phenomenology

So far I have offered some thoughts on the method of Barnes’ book, suggesting that there are problems with this approach, including obscuring the diversity (of goal, target concept, and focus) of the views discussed. I have particular concerns about the attempt to position phenomenological theories as one type of normative theory of health (Chapter One, section 4). It is a misunderstanding, I think, to position phenomenological theories as normative theories of health, and an error to describe them as subjective. I think they are neither and here’s why. Without the distinction phenomenology offers, between disease and illness, one may think, as Barnes suggests, that phenomenological theories are subjective “insofar as they make health a matter of personal felt experience rather than an objective biological condition” (p.51). However, phenomenological views do not suggest that there is no objective state which constitutes disease (or pathology, to use Barnes’ helpful term), but that to understand why we care so much about disease we need to understand the illness experiences it gives rise to. If disease didn’t cause experiences of pain, fatigue, inability, and distress, we wouldn’t care about it as much as we do. This experiential feature is not the *only* component of that state that we care about, but it is certainly its most significant feature. The task of phenomenological theories is not to offer definitions of *disease*, but to study, describe, and articulate *illness as the lived experience of disease*. This can be done in many ways: we could phenomenologically study the shared features of illness (Toombs 1987), we could ask whether wellbeing is possible within the constraints of illness (Carel 2016, 2018), we could study experiences of illness across cultures and times (strands of medical anthropology and medical history do that), and so on. It is a research program more than a theory. And it certainly does not purport to, or need to, offer a definition of disease. Barnes does not think this is a problem of this approach, as she notes in footnote 62 (p.52).

It is therefore not a problem for this set of views to have states in which there is disease with no illness (early stages of coronary disease, for example), or illness without disease (perhaps mild depression which is not currently known to correlate to any brain state is an example of that). Even in cases where there is both disease and an illness experience of that disease the correlation isn’t predetermined or fixed. This allows the view to easily contain – and indeed offer an explanation for – the common case in which two individuals suffer from the same disease in the same stage (e.g. stage 1 breast cancer) but live it (experience it) entirely differently. One can have a pessimistic, painful, distressed illness experience. The other can experience the very same disease in wildly different ways (maybe they minimise it, or laugh at it, or feel it is a spiritual journey – these are all possible ways to experience one’s disease). What phenomenology is searching for are ways to allow such vastly divergent experiences to not be fixed or standardised in the same way disease states are. It enables unique illness experiences to have space, and for the presence of what modern medicine calls ‘the person’ (that of ‘person-centred care’). At the end of the day, although this is not the only component in this complex thing we call ‘health’, it’s certainly the component that matters the most.

Finally, a phenomenological account enables us to also make space for moral, social, existential, spiritual, interpersonal and other dimensions of illness to be noticed and expressed; dimensions that are often absent from medical discourse and from medical notes. What we can particularly appreciate about the phenomenological approach is that it offers a robust and nuanced set of concepts with which to understand experiences of illness without reducing them to either the Scylla of reductive medical jargon or the Charybdis of rigid social scripts (illness is terrible; ill person as hero; illness as journey/ gift/ mission and so on).

Applying Heidegger’s (1962) notion of ‘being in the world’ (*In der Welt Sein*) to illness captures how everything changes in the life of the ill person, because it views their ways of being within a particular world, with others (*mit-Welt*), and with its specific environment (*Umwelt*) as interconnected (Carel 2016, 2018). Without Merleau-Ponty’s (2012) ‘third way’ conceiving of the person as a body-subject we would find it hard to articulate the inseparableness of one’s body from one’s experience, life, and being, and hence how any change to embodiment has global impact on the person (Carel 2016, 2018). Illness changes one’s world as a whole and within it one’s social relations, one’s relationship to time, space, the environment (the ‘geography of illness’). The role of phenomenology of illness is to work this out in detail.

Now of course other things can change our being in the world and our relationships to ourselves, our environment and to others. Illness experiences are akin to, in some ways, and on a continuum with, other ways of feeling discomfited by one’s body. Barnes presents this as a criticism of phenomenological approaches. But *contra* Barnes, this continuum isn’t a way of showing that phenomenological descriptions are too promiscuous, but rather that they share much with these other kinds of body-originating forms of dis-ease. This allows the political dimension of illness and of discrimination against ill persons to be made visible and positions illness (or better, pathophobia, to use Ian James Kidd’s (2019) useful term) as standing shoulder to shoulder with sexism, racism, ablism and other forms of discrimination and prejudice. Moreover, they also intersect in interesting and potentially fruitful ways.

A phenomenological approach to illness can also enable viewing experiences of ill health as sharing important commonalities with other types of personal adversity. This has several advantages, including a broader contextual understanding of illness as one kind of negative experience that is largely negative but also has edifying potential (Carel 2021a; Kidd 2012). It also allows us to see the social and political aspects of illness, in particular from the perspective of social justice. Epistemic injustice in health care is a current research project^1^ investigating such intersections, and aiming to give expression to the moral and political ways in which such stereotypes disable and harm ill persons (Kidd and Carel 2016).

Phenomenological analyses can reveal (1) that illness is on a continuum with other ways in which bodies serve as the basis for mistreatment, stigma, prejudice and injustice. And (2) that ill health is also part of a broader set of life events and situations we can call more generally ‘adversity’ and that our responses to adversity disclose robust connections between illness and other kinds of personal and collective harm, trauma and suffering. It is those political and existential dimensions of illness and its affinity with other kinds of body-based discrimination, and with other types of adversity that are important to develop. Phenomenological approaches enable us to do that in a way that definitional accounts of health do not. Phenomenology is not just the generation of accounts of idiosyncratic experiences and narratives. It is the articulation and discerning of experience, which can then be positioned within a framework of phenomenological concepts which give space to idiosyncrasies but remain embedded in shared aspects of human experience.

Without the distinction between disease and illness, the phenomenological account of illness does not make sense. This is important because a. it preserves the connection between feeling bad and having something objectively wrong with your body or mind (so not any malaise counts as an illness); and b. because that shows that the phenomenological project is not to define disease – in fact, it isn’t at all to do with disease (so saying it’s a ‘slightly different project’ to Boorse’s is an understatement). Barnes’ objection that not all pathology has symptoms is therefore fully and pre-emptively addressed by the distinction between disease and illness.

Barnes further suggests that “feeling at home” is what it means to be healthy for phenomenologists. I don’t think that’s right. It means feeling at home in terms of one’s bodily function, bodily trust, and bodily ability. It does not mean that the person who feels at home with respect to their health also feels at home emotionally, spiritually, socially and so on. Feeling at home in one’s body is much more complex; phenomenology tries to resist simple equations such as this. Illness can give rise to feelings of bodily alienation, but it may not: one can feel ‘ill, but well.’ And feelings of bodily alienation could stem from other sources too, as Barnes rightly points out. Bodily alienation is one common, but by no means universal, feature of illness experiences.

This also returns us to the idea that one can feel well within the broader context of chronic illness (Carel 2016). There is strong empirical evidence that one can feel well, whole, at home in the world, and so on even with very serious physical constraints. Disability activists like Harriet Johnson say similar things about disability (Johnson 2003). It’s not that the physical impairment isn’t there, is denied, ‘bright-sided,’ or idealised. Rather the phenomenological approach gives space to the possibilities of wellbeing, flourishing, and wellness within the constraints of illness, rather than closing off these possibilities to those who are ill. The goal is to open a space for ill and disabled persons to have a stab at the good life, rather than be automatically excluded from it on grounds of ill health alone (Carel 2016, Chapter 6).

A final comment on congenital illness: Barnes suggests that the contrast phenomenologists draw between healthy and ill lived experience is absent in cases of ill health that is congenital. This is a good point, to which there are two responses. First, the contrast is not essential for the feeling of disease, of being unwell, etc. One can feel unwell, *tout court*, their whole life and have a deep appreciation of the fact that they could have felt better. Second, social comparisons would still be there even in cases of congenital disease. Consider a young child with cystic fibrosis, which they have been ill with since birth. Even if they have never been healthy, such a child will be aware that they are missing out, unable to participate, and are subject to special restrictions, when compared to others. The contrast will still be salient but come from interpersonal comparisons, rather than from intrapersonal pre- and post-illness comparisons.

### How messy is the reality of health?

Finally, I want to pick up Barnes’ third point in her health redux of Fara’s view (Chapter Five section 3). It’s a good and important point that can be very useful. In many philosophical debates about disease borderline cases are discussed and used to show that one definition is better than a competing one, or to show problems with a particular account. That process is helpful in philosophy, but not in medicine, because precision is often not required in medicine. This may strike one as a counterintuitive claim, but I think it’s a core feature of clinical medicine and part of what makes it an art as much as a science. To use Barnes’ point about precision, philosophy requires very high levels of precision, both in its concepts and in its aims. But in other cases we can benefit from having imprecise aims and goals (p.240). In other contexts, including that of clinical medicine, this precision is not required. Philosophers sometimes suggest that because a cholesterol level of 4.8 is normal but 5 is high that means that the cutoff point is arbitrary, or imprecise. But I don’t think that’s right. Rather what we are seeing is a ballpark judgement: a clinician won’t be troubled by a cholesterol level of 5.1, but will prescribe statins at 5.5 or 6. Barnes’ point that precision is not always necessary is helpful in showing that the exacting standards of philosophy are not useful in a central case of talking about health, namely, in clinical medicine.

Continuing from that, it is not clear to me that what is messy is the reality – we can know very precisely that someone’s cholesterol level is 5. That part of medicine is straightforward science. The question is how to interpret that fact, what action to take as a result, and how this fact interacts with other factors, including the ill person’s own views and goals. And this is where medicine’s ‘art’ supplements the science. The problem isn’t that we need more precision – a cholesterol reading is precise. We need to pragmatically interpret that measurement and then decide on treatment options, policy, public health priorities, funding, and so on, and that’s the hard bit. I am not sure this means that health is a messy concept. It certainly is *complicated* and *complex* because of the multiple factors and dimensions to health, which are laid out beautifully in Barnes’ book. What is needed isn’t more precision, but finding better ways to work with patients to jointly choose the most suitable, effective, useful, interpretation of objective medical facts about them and what they want to do about them. And that is, I think, the important message of *Health Problems*.

## Footnotes

1 Funded by Wellcome. See: www.epistemicinjusticeinhealthcare.org [accessed on 10.4.2024].
